# Emergency Department Management of Common End-of-Life and Palliative Care Symptoms: Three Cases

**DOI:** 10.7759/cureus.53538

**Published:** 2024-02-04

**Authors:** Alice Chang, James Espinosa, Alan Lucerna

**Affiliations:** 1 Emergency Medicine, Jefferson Health New Jersey, Stratford, USA

**Keywords:** symptom management at the end of life, dyspnea management at the end of life, pain management at the end of life, end-of-life care, palliative care

## Abstract

The emergency department (ED) is at times the only place where patients can turn for symptom relief. Patients of all ages may turn to the ED for help with the management of end-of-life (EOL) and palliative care (PC) symptoms. Emergency medicine (EM) is a specialty that manages disease-directed treatment for a variety of acute conditions. In contrast, EOL and PC are focused on improving quality of life. Patients with serious illness, even hospice patients, present to the ED in increasing numbers for symptom management. It has become essential for emergency physicians to care for patients who are not seeking life-sustaining measures but instead need quality-of-life interventions. The development of a clear, concise review of the most common acute symptoms can provide a framework for EM physicians to adequately address the needs of patients at the EOL. Here, we discuss three cases that highlight the management of five of the most common EOL and PC presentations to the ED.

## Introduction

Patients with end-stage diseases, such as terminal cancer, are particularly prone to symptoms that are challenging to manage in an outpatient setting [1,2]. These patients, from home or hospice, present to the ED in increasing numbers for symptom management [3]. A survey of emergency physicians suggested that there may be a lack of training and guidelines for emergency physicians for the symptomatic management of end-of-life (EOL) care [4]. A study in Ontario indicated that 26% of advanced cancer patients visited the ED during the final two weeks of their lives [5]. Another study shows that 24% of terminal patients who presented to the ED in the last months of their lives cited pain as the primary reason, followed by shortness of breath at 21.5% [6]. Even those who have established palliative care (PC) involvement may present to the ED, often precipitated by the distress of family members who cannot adequately manage their symptoms [2]. There is a growing recognition that initiating comfort care in the ED yields numerous advantages [7]. The transition from curative to non-curative symptom management can be immediate. Emergency physicians need to skillfully adapt their care to respect the goals and values of the patients and their families.

In a study, emergency medicine (EM) residents ranked pain and dyspnea management as 1st and 3rd, respectively, in terms of important topics for a PC curriculum [4]. EM residents reported minimal training in pain management and managing hospice patients [4]. Numerous studies have identified common symptoms at the EOL requiring medical intervention - pain, dyspnea, delirium, nausea, and constipation [8,9]. However, there is a lack of structured, practical guidelines and education for emergency physicians on managing EOL symptoms. Patients with terminal illness can have burdensome symptoms that are difficult to manage. Studies have identified nausea, constipation, dyspnea, pain control, and delirium as common PC and EOL symptoms [8,9]. These are symptoms that are commonly seen in the ED population but may require different approaches and treatments in palliative patients.

This paper was presented as a poster presentation at the Canadian Hospice Palliative Care Association Conference, held in Ottawa, Canada on October 12, 2023.

## Case presentation

Case 1

A 47-year-old woman with a past medical history of breast cancer was sent to the ED by her oncologist for intractable nausea and vomiting. The patient was on chemotherapy. She had been taking dissolvable ondansetron and oral prochlorperazine with no relief. Her oncologist was concerned she was becoming dehydrated. The patient also complained of constipation and no bowel movement in three days. She was noted to be taking 10 mg of oxycodone every six hours for cancer pain. In the ED, the patient appeared to be clinically dehydrated, with dry mucous membranes and mild tachycardia at a heart rate of 110 beats per minute. Laboratory studies, including a complete blood count and basic metabolic panel, were within normal limits. A computed tomography (CT) scan of the abdomen and pelvis did not reveal small bowel obstruction or other acute findings. The patient was given 10 mg intravenous (IV) metoclopramide with some improvement but had persistent nausea. The patient was subsequently given olanzapine 5 mg orally and her symptoms resolved within an hour of the administration of olanzapine. She was also given two liters of IV normal saline bolus. The patient was given a glycerin suppository as well as methylnaltrexone 12 mg subcutaneously for her constipation. The patient subsequently had a bowel movement. The patient was then able to tolerate fluids orally and was discharged from the ED within five hours of arrival with a prescription for oral metoclopramide as needed as well as a daily bowel regimen. The case is from March 2023.

Case 2

An 87-year-old male on hospice care for terminal colon cancer presented to the ED from a nursing facility for dyspnea. The patient presented with a signed Physician Orders for Life-Sustaining Treatment form documenting "do not resuscitate, do not intubate, and do not hospitalize." The patient was awake and alert but appeared fatigued with labored breathing. He had copious, clear oral secretions with normal breath sounds on lung examination. The patient was then given atropine 0.5 mg IV after suctioning failed to control his secretions, with only mild improvement. However, he continued to have dyspnea. The patient's oxygen saturation was 97% on room air. Lab work, including a complete blood count, basic metabolic panel, and urinalysis, was within normal limits. A CT angiogram of the chest was negative for pulmonary embolism, pneumonia, or other acute findings. The patient's dyspnea was felt to be secondary to pain. The patient’s rescue dose for breakthrough pain dose was calculated as 10% of his total daily opioid dose. It was then converted to the IV equivalent and the patient was given 50% of the calculated dose for his dyspnea. The patient’s symptoms resolved in the ED after a six-hour stay, and he was discharged back to the nursing facility with outpatient PC follow-up. The case is from June 2022.

Case 3

A 70-year-old female with a history of multiple myeloma presented to the ED from home for intractable back pain and delirium. The patient’s family stated that she had recently enrolled in a PC program. They reported that the patient had back pain due to lesions of her lumbar spine and takes 10 mg of oral morphine every six hours. The family also stated that the patient has been more confused than usual. Lab work and CT of the head and lumbar spine did not show any acute process. The patient was given her breakthrough pain medication based on her total daily morphine dose, after converting it to IV form. The patient’s symptoms were re-evaluated, and repeated doses were given as needed. While in the ED, the patient became more confused and agitated, requiring chemical sedation. The patient was given haloperidol and was observed in the ED over a six-hour period. The patient’s mentation returned to baseline and had adequate pain control. The patient was discharged home with family. The case is from September 2022.

## Discussion

**Figure 1:**
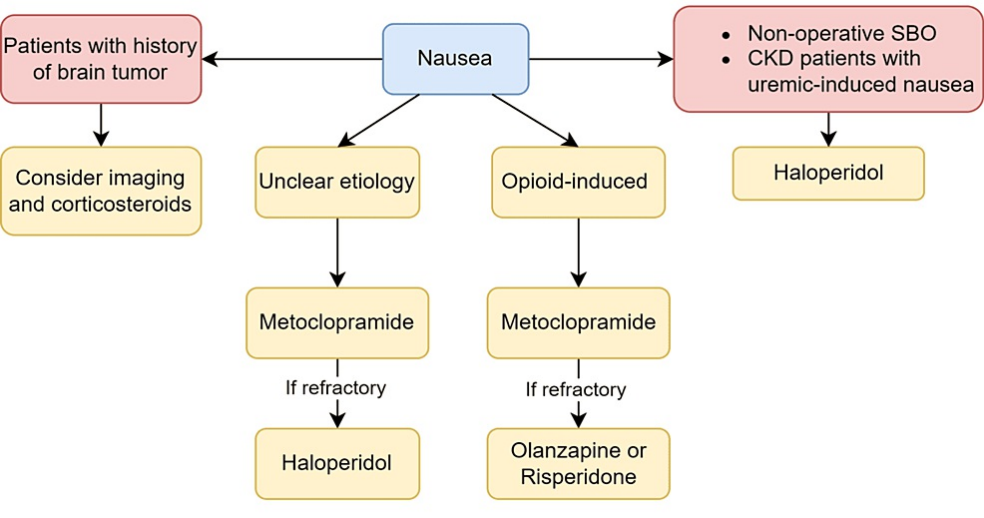
Management of nausea in palliative care and end-of-life patients. SBO: small bowel obstruction; CKD: chronic kidney disease.

The patient in case 1 presented with nausea. Nausea is another common symptom in PC and EOL patients, caused by a variety of factors, including pain, medication side effects, and disease progression. The workup to evaluate for the etiology of nausea in palliative patients should be purposefully focused on the history and physical examination. In advanced diseases, the burden of diagnostic lab work and imaging may outweigh the benefits. Certain etiologies common in PC patients require more specified management. PC patients are more commonly on opiates for pain control. Opioids have three potentially nausea-inducing mechanisms; it has a direct effect on the chemoreceptor trigger zone, enhanced vestibular sensitivity, and delayed gastric emptying [10]. For patients with opioid-induced nausea, metoclopramide is the first-line treatment [11]. For nausea-refractory cases, atypical antipsychotics, such as olanzapine or risperidone, can be initiated [12]. For patients with brain tumors, corticosteroids are first-line therapy, given their ability to decrease cerebral edema [12]. If suspected, imaging should be considered, as it will provide directed symptom management. Studies have shown that metoclopramide is a reasonable initial choice for nausea management of palliative patients with unclear etiology [11]. Haloperidol is often used as a second-line drug for those who have contraindications or refractory to metoclopramide [11]. It is also first-line therapy for patients with non-operable bowel obstruction as well as chronic kidney disease patients with uremic-induced nausea [11]. Olanzapine has also been shown in studies to be an effective alternative or adjunct therapy for advanced cancer patients with no clear etiology for nausea and vomiting (Figure 1) [12].

**Figure 2:**
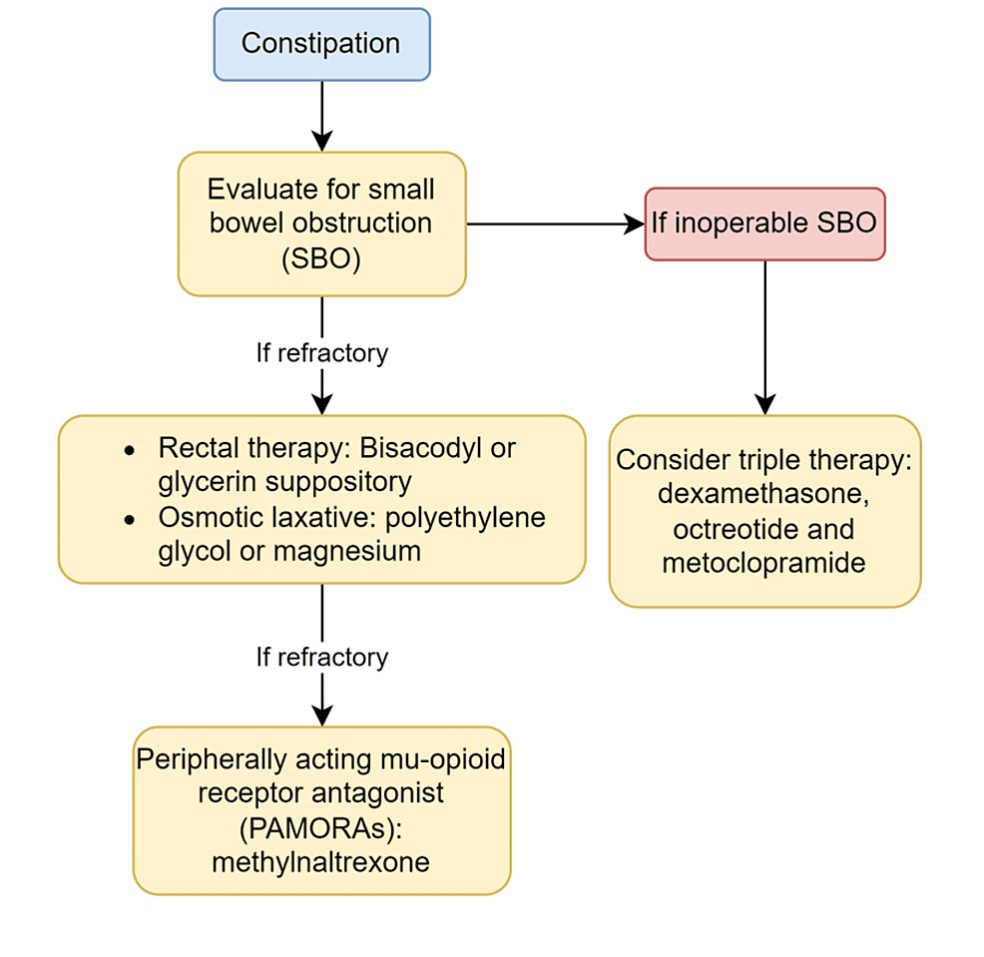
Management of constipation in palliative care and end-of-life patients. SBO: small bowel obstruction.

In addition to nausea, the patient in case one presented with constipation. Constipation is a common side effect in patients with chronic opioid use. Patients are often on daily bowel regimens, such as senna. Unfortunately, patients can have refractory opioid-induced constipation. These patients should be first evaluated for bowel obstruction as appropriate based on the history and physical examination and with imaging as appropriate. If there is no evidence of bowel obstruction, patients with opioid-induced constipation despite prophylaxis can be treated with rectal therapy, such as bisacodyl or glycerin suppository, or an osmotic laxative, such as polyethylene glycol or magnesium. As illustrated in case 1, in patients with refractory opioid-induced constipation, peripherally acting mu-opioid receptor antagonists (PAMORAs), such as methylnaltrexone, are effective agents for ED use [13]. The use of glucocorticoids for malignant bowel obstruction can be considered, but the data to support efficacy are relatively weak [14]. However, studies have shown that when glucocorticoids are added to the regimen with antisecretory therapy with an antiemetic, they provide synergic effects given the different mechanisms of action [14]. A study showed that triple therapy with dexamethasone, octreotide, and metoclopramide led to higher rates of de-obstruction in patients with inoperable bowel obstruction (Figure 2) [15].

**Figure 3:**
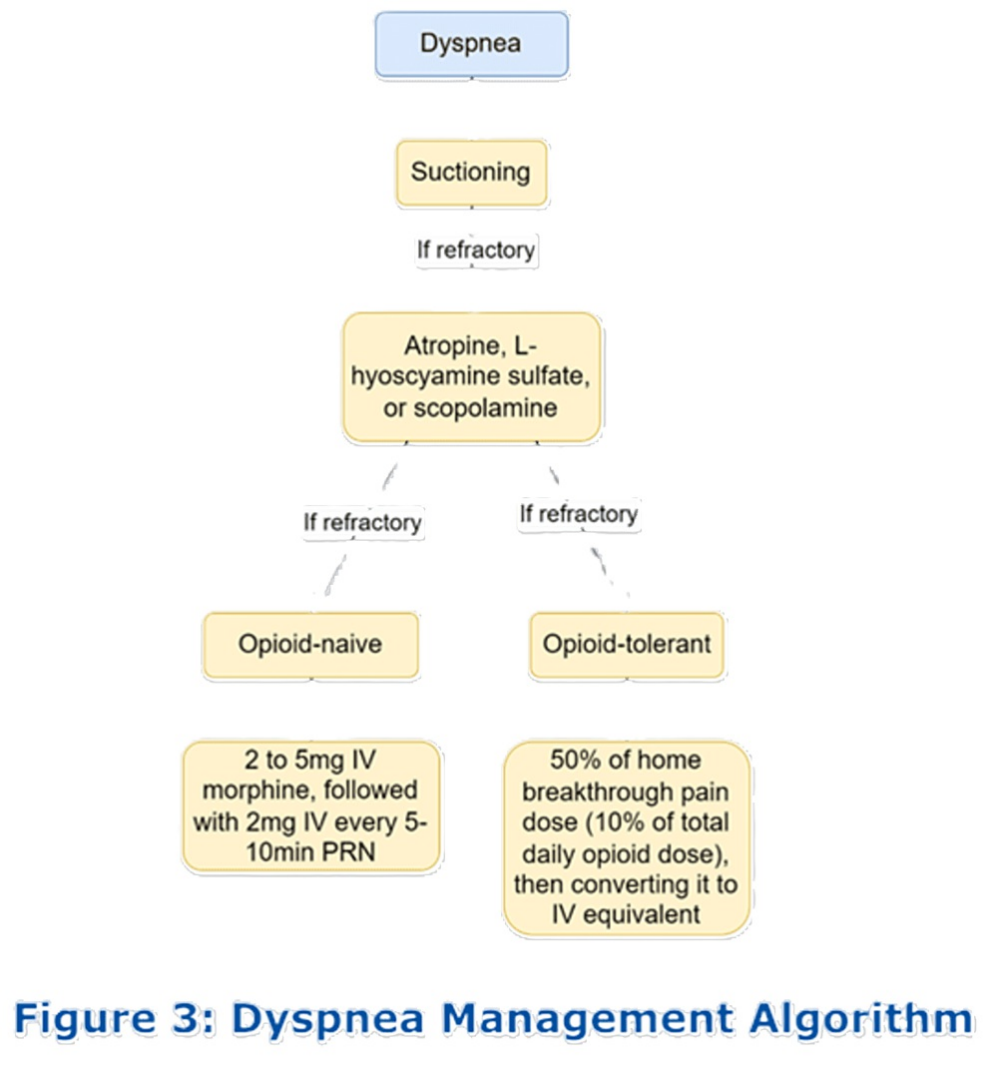
Management of dyspnea in palliative care and end-of-life patients. PRN: pro re nata.

The patient in case 2 presented with dyspnea. Dyspnea is a common experience for patients near death [16]. Respiration rate can be normal until close to death and is not a reliable indicator of the discomfort they may be experiencing [6]. As with any other patient, the first step is to identify the treatable causes. The mnemonic BREATH can be used to evaluate common causes of dyspnea at the end of life [6]. It stands for bronchospasm, rales, effusions, airway obstruction, thick secretions, and hemoglobin low. The “death rattle,” often related to fluid collection in the airway, can be particularly startling for caretakers. Patients and their families can be comforted by the knowledge that it may not be associated with dyspnea or the sensation of drowning. These patients can benefit from suctioning or pharmaceutical interventions, such as atropine, L-hyoscyamine sulfate, or scopolamine [17]. However, if dyspnea continues after the correction of treatable causes, palliative treatments should be enforced. For opioid-naïve patients, 2-5 mg IV morphine provides relief for most, followed up with 2 mg IV morphine every five to 10 minutes as needed [18]. For patients already taking opioids, dyspnea can be treated by calculating 50% of their home breakthrough pain medication dose and then converting it to an IV equivalent dose. The appropriate dose for breakthrough pain is 10% of the total daily opioid dose (Figure 3) [18].

**Figure 4:**
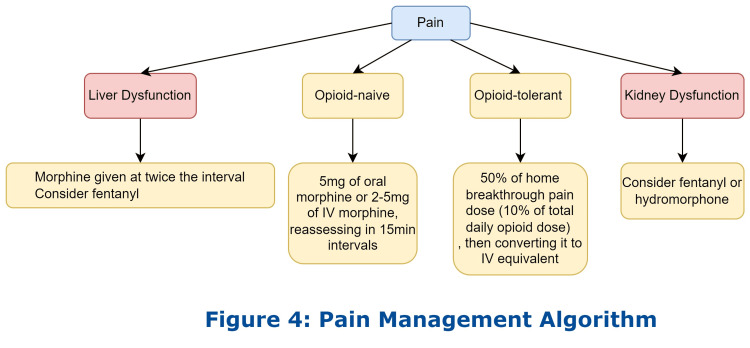
Management of pain in palliative care and end-of-life patients.

Pain control is one of the most common reasons why PC patients present to the ED, as illustrated by the patient in case 3. PC patients should have a home regimen with oral pain medications but often present to the ED if it is insufficient with breakthrough pain. Most PC patients are prescribed oral morphine, oral oxycodone, opioid patches, and subcutaneous opiates for home/hospice pain management when non-steroidal or other non-narcotic pain medications are not effective [19]. For opioid-naïve patients, the National Comprehensive Cancer Network recommends initial doses of 5-15 mg of oral morphine or 2-5 mg of IV morphine, reassessing at 15-minute intervals [19]. For patients already on opioids and who have developed tolerance, treatment for acute breakthrough pain can be calculated as 5% to 20% of the total opioids taken in a 24-hour period, given orally or converted to an intravenous equivalent if needed. For example, if a patient takes 100 mg morphine orally every 12 hours, the total 24-hour dose would be 200 mg morphine orally. If the breakthrough pain dose is calculated at 10%, the patient would be given 20 mg morphine orally or about 6 mg morphine IV when converted to parental form. The intravenous morphine to oral morphine ratio is 1:3, and the intravenous morphine to oral oxycodone ratio is 1:2 [20]. For patients with significant liver dysfunction, morphine should be given at a lower dose spaced out to twice the usual dosage interval to avoid accumulation. Fentanyl should be considered as it is metabolized to non-toxic metabolites [21]. For patients with kidney dysfunction, opioids that are renally excreted, such as morphine, should be avoided or used with caution, and fentanyl or hydromorphone should be considered as an alternative treatment [21]. Pain medication for chronic pain related to terminal conditions should be provided on a scheduled basis instead of as needed. Transdermal or other long-acting oral agents should be considered for pain control in patients with chronic pain (Figure 4) [22].

**Figure 5:**
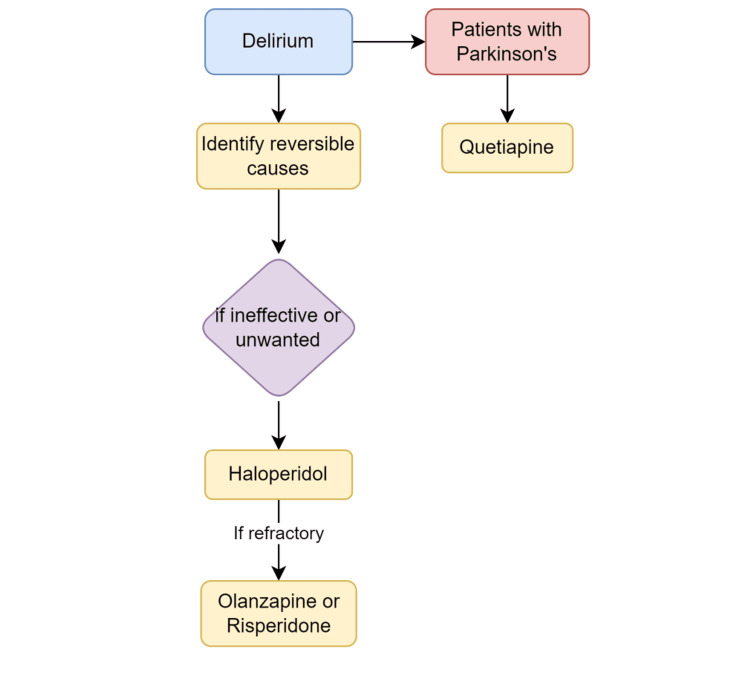
Management of delirium in palliative care and end-of-life patients.

Delirium in palliative patients not only causes distress for the patient but also for their families, prompting them to visit the ED, as is seen in case 3. The primary goal of treating delirium is identifying reversible causes. PC patients often have a complex medical history, and polypharmacy should be considered. This can be addressed by evaluating a patient's medication list and initiating a rotation of opioid medications. Pain is a key cause of delirium and should be addressed if appropriate [23]. If treatment of the underlying etiology is ineffective or unwanted, antipsychotics such as haloperidol are the first-line treatment for delirium [15]. Alternatively, olanzapine and risperidone are also effective treatments [15]. For patients with Parkinson's disease, quetiapine would be the preferred antipsychotic [15]. Benzodiazepines such as lorazepam should be avoided if possible, as they may exacerbate symptoms (Figure 5) [24].

Effective symptom management of PC patients in the ED is crucial for several reasons. PC patients are a vulnerable population with complex needs that require specialized care. These patients often have multiple comorbidities, are at increased risk of complications, and may have limited functional capacity. Effective symptom management can improve the patient's quality of life and prevent unnecessary suffering. The ED is often the first point of contact for patients with advanced illness and may be their only access to health care outside of regular clinic hours. Prompt and effective symptom management can help avoid unnecessary hospital admissions, reduce healthcare costs, and improve patient outcomes.

Barriers exist to effective symptom management in PC and EOL patients. Although the common symptoms PC patients experience at the EOL are often seen in the ED, they require more directed management in this population. Additionally, there are several barriers to adequately addressing these symptoms in the ED. One study suggested that emergency physicians lack training in symptom management specific to PC patients [4]. This can impede the proper management of EOL symptoms. For example, a common obstacle to opioid use for dyspnea is the fear of respiratory depression from providers. However, studies have demonstrated that titrated administration of opioids provides significant relief of symptoms without evidence of clinically significant respiratory depression or decreased oxygen saturation [25]. Pain management at the EOL can be particularly complex, as factors beyond the underlying medical conditions can influence the treatment that patients receive. For this reason, the palliative care team and the pain team often work together in patient management. For patients and families, there is the fear that pain medication can take away independence and diminish mental capacity, as well as the fear of addiction [6].

A clear understanding of EOL management and structured protocols for treating EOL symptoms in the ED can provide EM physicians with greater confidence in providing PC and EOL care [26]. One effective approach to providing guidelines for ED providers in palliative symptom management is through simple and reliable protocols, especially focusing on the five most common symptoms. Studies have shown that stepwise protocols for symptom management can be highly effective. For example, the World Health Organization (WHO) analgesic ladder protocol proposed in 1986 has been shown to reduce morbidity due to pain in 70% to 80% of cancer patients even in recent studies [27,28]. Another study demonstrated that an EOL protocol initiated in the ED, with specific guidelines on how to manage common EOL symptoms, increased the use of opioids for dyspnea and hyoscyamine sulfate for secretions, as well as improved patient satisfaction with care [16]. These findings highlight that practical and easily implementable protocols can significantly improve symptom management in the ED for PC patients [29].

## Conclusions

Optimizing patient care and improving the quality of life for PC and EOL care patients in the ED requires effective symptom management. PC and EOL patients may seek help from the ED for symptom control, even if they do not wish to undergo aggressive measures. While pain, dyspnea, and other PC and EOL care symptoms are commonly encountered in the ED, it is crucial for emergency physicians to recognize that managing these symptoms in such patients often requires specific approaches. Practical and effective implementations, such as palliative-specific symptom management protocols and standardized order sets can have an immediate impact on the care that is provided to the most vulnerable patients.
